# New Possibilities for Testing the Service Life of Magnetic Contacts

**DOI:** 10.3390/mi12050479

**Published:** 2021-04-22

**Authors:** Martin Boroš, Andrej Veľas, Zuzana Zvaková, Viktor Šoltés

**Affiliations:** Department of Security Management, Faculty of Security Engineering, University of Zilina, Univerzitna 8215/1, 010-26 Zilina, Slovakia; andrej.velas@fbi.uniza.sk (A.V.); zuzana.zvakova@fbi.uniza.sk (Z.Z.); viktor.soltes@fbi.uniza.sk (V.Š.)

**Keywords:** magnetic contacts, torque, the calculation in memory, automation

## Abstract

Magnetic contacts we could define as a switching device used in transport structures such as a tunnel, to which the manufacturer prescribes a certain number of closures within its lifetime, during which they should operate flawlessly. Verification of the data provided by the manufacturer is time-consuming and physically demanding due to the data being large in number. For this reason, we developed a test device using torque in the research of magnetic contacts, which greatly automates the whole process and thus eliminates human error. The test device can use internal memory to calculate the number of closures of magnetic contacts and then transmit the digitized data. The test device is registered as an industrial utility model and can be used to test any magnetic contacts.

## 1. Introduction

Reliability is a natural property of products to perform the activities for which they were created, under predefined conditions and for a defined period. Reliability can be assessed for almost all devices in different types of industries. The parameter determining the service life of the equipment is also connected with reliability. We can divide the service life into two groups. One group consists of devices that define the reliability of time, i.e., the time during which the manufacturer declares their correct functionality, such as LED bulbs for which the manufacturer declares proper functionality somewhere at 50,000 h of use for lighting. The second group consists of switching devices in which the service life is defined in terms of the maximum number of switchings, such as conventional light bulbs for which the manufacturers declare the service life somewhere at the level of 10,000 switchings. In practice, this means that in the case of the first option, the LED bulb should stop shining after the time specified by the manufacturer, and in the case of the second option, if we turn the bulb off and on in half the number specified by the manufacturer, it should also stop shining or lighting up [1,2,3,4]. The service life of electrical equipment, in particular, has been a very inflected concept in recent years, as some are convinced that manufacturers purposefully install a time-memory component in equipment that is activated after a certain period of use, usually after the warranty period.

An exception from the point of view of reliability is also the electrical devices or components forming an electrical security system designed to identify and detect the presence or attempt of an intruder to enter the building. Even in this case, we could apply the above distribution of life because while the passive infrared detector must be able to detect the guarded area for a long time, the magnetic contact used to secure windows and doors has a predefined value of the number of closures from the manufacturer [5,6,7,8].

**Figure 1:**
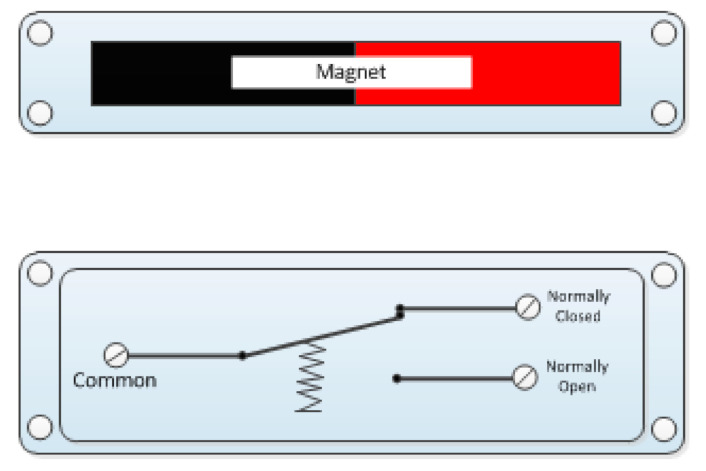
Principle of operation of magnetic contact [7].

Magnetic contacts are used in almost every industry for various purposes, for example, to detect the opening of switch cabinets and switchboards in electronics, as an opening mechanism in keyless furniture designs, in the toy industry, and many others. From the point of view of security, as mentioned, they have their fixed place in the electrical security system where they are used as opening detectors, i.e., they sound an alarm in case of unauthorized opening of windows, doors, gates, or blinds or shutters on windows. Depending on the nature and requirements of the secured object, it is possible to create complete protection from magnetic contacts by fitting them on all doors and windows. The magnetic contacts are formed by a permanent magnet and a contact, also called a reed contact. The reed contact consists of a sealed glass tube filled with a protective atmosphere in which two ferromagnetic contacts are placed (Figure 1). The magnet is installed on a moving part of a window or door and a reed contact on a fixed part, i.e., a door frame or frame. At the moment when the permanent magnet is close to the tongue contact, it is in the basic state, usually closed, due to the magnetic field of the permanent magnet. When the magnet is moved away, for example, due to the opening of a door or window, the magnetic field affecting the tongue contact weakens, and the contact changes its state to the opposite. If the contact is of the normally closed type, it opens, and if it is of the normally open type, it closes [7,9,10,11,12].

The reliability or correct functionality of the magnetic contacts can be tested according to predefined possibilities, as specified in more detail in the European technical standard with the type designation EN 50131-2-6 [13]. The technical standard prescribes several possibilities, or types of tests where the main one is the resistance of the magnetic field to the external magnetic field generated by the parasitic magnet. In this case, it is determined whether the electric security system correctly evaluates the attempt to disrupt or sabotage the system. In addition to the parasitic magnet test, the technical standard defines other tests, such as the basic detection function test in which the values of closing and opening of the magnetic contact are verified and then compared with those specified by the manufacturer. However, these and other types of tests have one basic disadvantage/disadvantage and therefore, given the possibilities of today’s market, they are relatively strict and do not take into account the possibilities of various commonly available magnets [10,13]. The second mentioned test, or the basic detection function, could be described as a direct verification of the reliability of the magnetic contact. This is because it takes place in a slow, gradual, and simulated movement from the closed position of the magnetic contact, with the magnet facing away from each other, thus determining the actual value of the opening. Subsequently, the whole process is repeated, but in the opposite direction, which determines the value of closing the magnetic contact. The same procedure can be performed in the case of the oblique direction of the permanent magnet, as it should be able to work in all directions of movement. These types of tests are relatively demanding and in many cases even lengthy, which increases the risk of error of the human factor, i.e., the person performing the tests themselves. One of the basic and challenging tasks is to accurately determine the value of individual distances. To avoid errors and inaccuracies in the test, it is recommended that graph paper be used to determine the values better and faster. To know when the magnetic contact closes so that it is activated, it is necessary to connect a signaling device to it. In the absence of a signaling device, it is possible to use a complete installation of an electrical security system with a control panel, with which we can know when the contact was activated [14,15,16,17].

Within the professional community, it is possible to observe several research activities aimed at measuring the reliability of magnetic contacts, but in all cases they represent only a partial part of the research. For example, Gong and his team focused on measuring the sorting of magnets in factory production, in which they pointed to the lack of homogeneity of the magnetic field. In one of their studies, the Carus team focused on improving the efficiency of electric drives using permanent magnets, within which the goal was to define a uniform procedure for measuring the impact on control algorithms [11,18,19].

Despite measuring and testing the reliability of magnetic contacts, it is always a question of using magnetic contacts, as mentioned, and not the switching capacity itself. Therefore we decided to research the total magnetic contacts for security needs to pay attention to their actual switching functions, and within it and its recurrence.

As mentioned above, magnetic contacts are one of the basic components of electrical security systems designed to primarily protect the building envelope. To indicate the danger, they use the influence of magnetic induction, by which the permanent part is attracted to the reed contact, which is currently supplemented by other electronics, such as an LED designed to indicate the closing/opening of the magnetic contact. The reliability of magnetic contact from a safety point of view is understood as to whether the opening and the opening of the window or door leaf caused by it is detected, as the magnetic contact needs to be supplemented with components in other layers of protection, such as spatial or object protection, for the complex security of the building. It is also highly important to use mechanical means of restraint to make it more difficult for the intruder to enter and escape from the secured object. It is necessary to realize that even the highest quality component of the electrical security system can be surpassed, and this depends only on the equipment and skills of the intruder. Therefore, in the professional and public sphere, we more often encounter the term probability, and if we consider a comprehensive assessment of the secured object, we use the term cumulative probability of intruder detection [20]. The cumulative probability of intruder detection is calculated using Formula (1) [6].

(1) $$Pcp=[1-\prod_{i=1}^{n}\left(1-Pd\right)]*Pats*Pf*Phf$$

where *Pcp* is the cumulative probability of intruder detection, *n* is the number of detection zones of the object, *Pd* is the probability of detection by active elements (magnetic contacts, passive infrared detectors or PID), *Pats* is the probability of transmitting intrusion information to the monitoring center, *Pf* is the probability of a fault-free state of the system, and *Phf* is the probability of human factor failure (guard, technician, system administrator).

To express the correct value of the cumulative probability, it is necessary to know as many input parameters as possible. In this case, it is the reliability of individual active elements installed in the building, i.e., detectors, which are designed to detect the intruder. As this is a large-scale issue, it is necessary to address it in the long term and extensively. In terms of time, such large-scale measurements could take several years if carried out by a single research team. Therefore, it is appropriate to divide the research activities related to the experimental measurements between several research teams that can work in parallel. As part of testing the reliability of individual components, data for PID [21] and transmission systems [22] were created based on experimental tests. PID detectors were chosen because of their frequent installation, as they are very often installed and they generate an alarm when detecting the movement of the intruder, and as a secondary reason because they are primarily used in spatial protection. We therefore chose the procedure in terms of selecting one main representative of the zone, for which we express the values of reliability. Another very important component is the magnetic contacts, which need to be tested as much as possible.

## 2. Materials and Methods

The overall research of the reliability of magnetic contacts from the point of view of safety could be divided into two groups of measurements. The first focuses on the accuracy of detection, activation, and deactivation of magnetic contact, and the second focuses on the life of magnetic contacts, i.e., the frequency of their connection or disconnection. In the case of the first group of measurements, we determined the exact procedure that we followed for all measurements as well as their repetitions. The basis was the division of this part into a part of closing, i.e., activation, and the time of expansion, i.e., deactivation of the magnetic contact. The essence of the measurement was the smooth movement of the magnet in the direction and against the axis of the magnetic field to express the approach and departure distance. After reaching the approach distance, a rest signal must be generated within the electrical security system, and after reaching the distance, an alarm signal must be generated [13,23]. The mentioned measurement procedure consisted of the following parts:

1. Becoming familiar with the magnetic contacts by studying the package leaflet from the manufacturer;
2. Performing the recovery and subsequent connection of magnetic contacts as well as other measuring components;
3. Realizing the measurements of the closing mode and opening mode;
4. Repeating each measurement 30 times for version 1 and 1000 times for version 2;
5. Calculating the arithmetic mean (*x*) of closing and opening of the magnetic contact using the relation $x=\frac{\sum_{i=1}^{n}x_{i}}{n}$, then rounding the values to whole numbers or halves upwards;
6. Comparing the results obtained with the data provided by the manufacturers.

The second group of measurements had essentially a very similar procedure, but the difference occurred in the case of point number 5, as the arithmetic mean was not calculated but only the total closing/opening of the magnetic contact was calculated. The aim of this type of measurement was not to know the working distance of the magnetic contact but its reliability at a preset distance. In the case of these measurements, we relied on a technical standard that allows the test requirements to be met with a tolerance of +/−10% [13].

**Figure 2:**
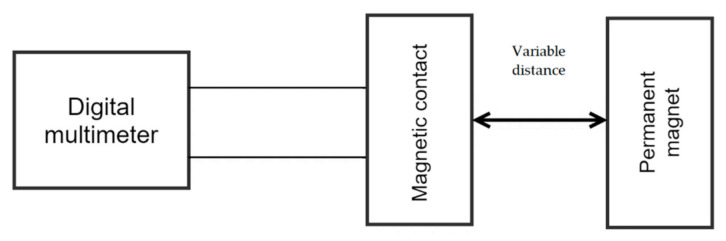
Circuit diagram of the testing version 1.

To facilitate the measurement process, we made a test device or test connection, which had the task of indicating the closing/opening of the magnetic contact. During the measurement, we even created multiple connections for a more comfortable and accurate result. Version 1 of test circuit 1 consisted of the use of a multimeter marked UNI-T UT70A, in which we used the possibility of circuit integrity, the so-called short-circuit probe, a ruler, and graph paper. The circuit diagram of test circuit 1 is shown in Figure 2.

After the implementation of the pilot 30 tests, we proceeded to the use of another measurement option, in which we decided to engage another sensation to identify the activation of the magnetic contact, namely sight. We therefore created a connection with an LED intended for visual inspection of the change in the state of the magnetic contact (version 2).

**Figure 3:**
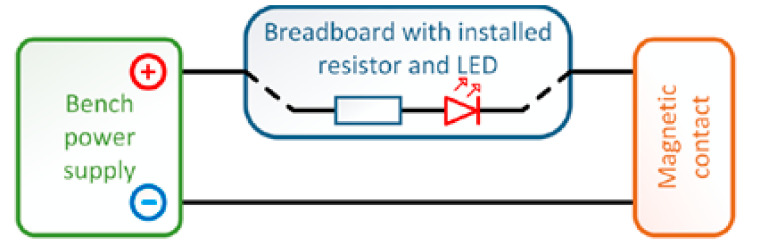
Circuit diagram of the testing version 2 [10].

Version 2 consisted of the electrical circuit, as shown in Figure 3, consisting of a laboratory power supply, a Yihua HY1503D, a 560 Ω resistor, and a color LED (red in our case). The output conductors, which were connected directly to the magnetic contact, switched the negative branch of the electrical circuit [23,24].

**Figure 4:**
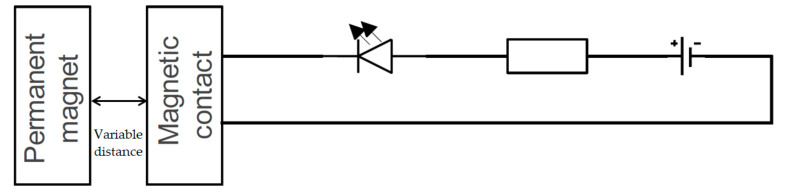
Circuit diagram of the testing version 3.

With the newly created test facility, we also conducted 30 pilot tests to determine which test facility is more accurate. However, the connection created was relatively impractical, mainly due to the large size of the laboratory source. Apart from the impracticality, from the source’s point of view, the measurement was done in an uncomfortable manner because we held both parts of the magnetic contact in our hands. We therefore decided to create an upgrade of the test equipment, which consisted of a fixed and a sliding part into which the individual parts of the magnetic contacts were installed. We made these parts from polymethyl methacrylate parts. Specifically, it was an “L” shaped profile in which a magnetic contact was installed. In addition to this, a contact field with a resistor and an LED was placed on the profile to indicate switching on and the power supply, which we solved using a backup power supply, consisting of two alkaline batteries marked LR03 with a nominal voltage of 1.5 V. A permanent magnet was attached to the second part, which is a moving part. This part was fully mobile and only added to the L profile. The advantage of this type was that the reading of the distance was instantaneous since on the L profile there was a scale of the ruler, the beginning of which is point 0, which was situated at the end of the fixed part and thus also of the magnetic contact. The circuit diagram of the improved version of the test circuit version 3; without the polymethyl methacrylate, the construction is shown in Figure 4 [13,23,25].

**Figure 5:**
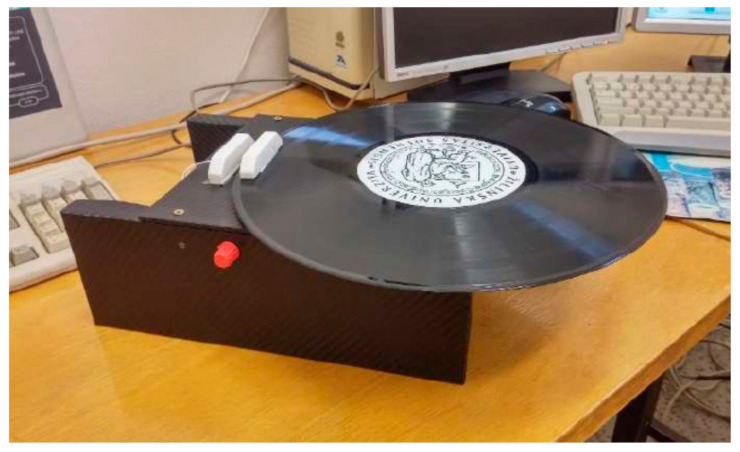
Magnetic contact life tester.

In the case of the second group of measurements, i.e., the lifetime of magnetic contacts, we devised a fully functional device that is protected by the Industrial Property Office as an industrial utility model. The essence of the test device is the possibility of performing a continuous rotary or oscillating movement, the basis of which is a twisting moment formed by a low-speed, motor drive. This was located in the lower part of the test device, together with the other technological part intended to express the number of repetitions, time recording, and the possibility of setting the speed, i.e., the speed of movement. With the help of the test device, it is possible to fully automate the measurement at a predetermined distance. This test device also consisted of two parts: the lower part with an electric motor, and the technical part was a fixed part to which the magnetic contact was attached. The movable part was formed by a plate that rotated around its center in the direction and speed asset. A permanent magnet was installed on its edge at the working distance of the magnetic contact. Ideally, the working distance was set approximately in the middle of the scale specified by the manufacturer or if the actual working distance from previous tests is known. Subsequently, the measurement was performed by successive rotations of the plate, and each time one circuit was performed, the given value was recorded in the internal memory of the device; also in a given number of rotations, the time is determined and whether the magnetic contact has been activated. The recorded data can then be downloaded from the test device to a portable device in the format of a file with the final .xlsx file, which can be edited in MS Excel and the measurement of which can be evaluated. In Figure 5, we can see the test device with the magnetic contact placed before starting the measurement [23,26].

The starting position for testing magnetic contacts was formed by pilot measurements, in which in addition to the measured values, we also evaluated the reliability of the given trial versions. Four commonly used magnetic contacts with type designations were selected for mutual comparison: Bestkey BP-1013, Bestkey BS-2013, SUNWAVE SD 8561, and USP 130SP.

These selected magnetic contacts were sequentially tested in all three versions of the measurement sets described in more detail. We decided to perform an extensive complex measurement focused on both the reliability and durability of magnetic contacts. In the initial first phase, we implemented a pilot of 30 measurements through all three test connections to determine the most accurate one. Subsequently, in the second phase, we performed 1000 repetitions with each magnetic contact using the most effective test circuit. In the final and third phase, we performed the lifetime measurement for 10,000 magnetic trials, for one magnetic contact, using the last described test device. For each measurement, the arithmetic mean of the measured values was determined, rounded up to whole numbers or halves. We obtained these values using the average function in MS Excel. We also determined the standard deviation for each measurement using the STDEV function in MS Excel. The overall results are shown in the following section.

## 3. Results

The first phase, as mentioned, was to test all the proposed test connections. The measurement was performed in two directions, first from point 0, a connected magnetic contact and a permanent magnet, and the subsequent removal of these parts, until the LED goes out and the distance is recorded. Subsequently, we moved the parts of the magnetic contact to a distance of 10 cm and gradually had them approach each other until the LED light came on. The values measured by the individual methods for the magnetic contacts tested are shown in Table 1 together with the standard deviation.

From the measured results, it is clear that the magnetic contacts do not correspond to the data given by the manufacturer in the technical documentation. In addition, in all four cases, we can observe the fact that the value of clamping itself is higher than the values given by the manufacturer. The opening values, which are a more important indicator than the closing ones, are in some cases even twice as high as the values given by the manufacturer [27].

Even the results in version 2 are not paradoxically better than those with version 1, but there is a slight shift to the values given by the manufacturer. On the positive side, the values of closing and opening decreased significantly by an order of 15 mm. The deviation can also be caused by the error rate of the human factor and the complexity of gripping the permanent magnet during measurement.

The final, third type of test device was a product in which the permanent magnet could be attached to the sliding part, thus increasing the comfort of measurement.

As we can see, the measured values using the test circuit version 3 are the closest to those specified by the manufacturer in the datasheet. We managed to measure, in the case of the magnetic contact Bestkey BS2013, the values corresponding to those from the datasheet. In some cases, for the Bestkey BR1013 we managed to reduce the value by 7 mm in case of opening, and in case of switching on we reached the level of the manufacturer. In other cases, the worst effect was the Sunwave magnetic contact, in which case we are far behind the values given by the manufacturer in this measurement.

A separate group of results is the values of the standard deviation, which in some cases are relatively high and almost at level 5. The smallest value is reached by the standard deviation in the case of the third version, so based on these data we can say that this version is the most reliable.

After completing the first phase followed by the second, we focused on 1000 repetitions using test circuit version 3. Gradually, we implemented all the repetitions for both the closing and opening of the magnetic contacts. Due to the complexity of the measurement from the point of view of attention, we took a break that lasted at least half an hour. The results, obtained together with the expression of the standard deviation, are shown in Table 2.

As we can see from the results given in Table 2, we were advised to achieve the average values, which fall within the intervals given by the manufacturer, using the test circuit version 3. The only discrepancy occurred in the case of the opening distance for the magnetic contact Bestkey BR1013, for which the distance is exceeded by 0.5 mm, i.e., a negligible distance. The values of the standard deviation are, except for one magnetic contact, at a very good level. According to the results, the problematic or faulty magnetic contact is SUNWAVE SD 8561. This magnetic contact has shown an appropriate degree of error or unreliability since the beginning of testing. However, it should be noted that despite the high degree of error, we measured either below or within the range specified by the manufacturer.

**Figure 6:**
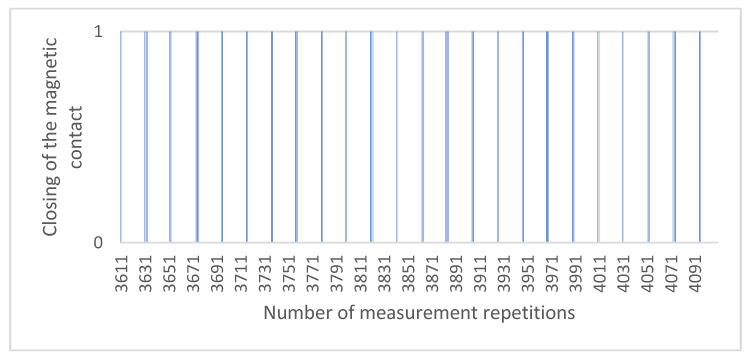
The data output of 500 repetitions from the test device (USP 130SP).

The last phase of testing was focused on experimental tests using an autonomous testing device, i.e., the complete construction of which is protected by the Industrial Property Office of the Slovak Republic. The device, as mentioned, uses the torque from the electric motor, the power of which and thus the torque can be adjusted using a potentiometer. During testing, the same conditions were ensured for each repetition, i.e., the distance as well as the speed of movement of the revolution was always identical for all repetitions. In our case, one turn lasted half a second, during which a step change occurred due to the closing of the magnetic contact. The recording was performed twice. In the first case it was a matter of counting one rotation of the rotating part about its axis, and in the second case, we counted whether the closing of the magnetic contact occurred. Subsequently, the data are recorded in the MS Excel software program, and it was therefore possible to clearly define in which rotation occurred and in which the closing of the magnetic contact did not occur. In the case of switching on, the value 1 was recorded, and if no switching was made, the value 0 was recorded. From these values, we plotted the switching on for magnetic contact USP 130SP shown in Figure 6. We gradually measured all magnetic contacts measured in the usual way in the previous expression. The measured values are given in Table 3. It should be noted that in these measurements, we used new pieces of magnetic contacts to avoid possible data distortion due to wear from previous measurements.

In the case of the last third phase, we decided to use the least erroneous USP 130SP. We subjected it to a load of similar 10,000 clamps at a predetermined working distance. For our measurement, we used the working distance achieved in the second phase, i.e., 22.5 mm. We measured the last phase on a test device, the export of which we subsequently evaluated in the MS Excel program. From the measured values we can state that the magnetic contact active in 9031 cases and in 969 not, which is within a tolerance of 10%, allowing within the technical standard. As the table with the data export would be confusing due to the size, we present the results in graphical form on Figure 6 for 500 randomly selected repetitions.

In Figure 6, we can see the step changes indicating the activation of the magnetic contact, where the initial position is 0 and the value 1 means the nonactivation of the magnetic contact in a given turn. The values in the figure do not reflect the duration of activation/non-activation of the magnetic contact; it only reflects the fact that the change has occurred. On the graph, 1 is specifically broken into 448 activations with a value of 0 and 52 nonactivations with a value of 1. The total measurement was 10,000 repetitions, and they lasted approximately two and a half hours.

From the results shown in Table 3, the deviations between the magnetic contacts are not at such a large level. Each of them was able to switch on (activate) approximately 9000 times, which represents a reliability of 90%. It should be noted that the activations represented a step change, a step duration of the magnetic pulse, for about half a second. With each rotation, the permanent magnet performed the same path and was always close to the reed contact for the same amount of time. The only difference in values could occur when activating the device.

## 4. Discussion

Pilot testing revealed several shortcomings, which we continuously eliminated, and with the help of the test connection version 3, we managed to realize 1000 repetitions of closing and opening of each tested magnetic contact. Together, in all phases of measurement, we performed 48,360 repetitions with two sets of four tested magnetic contacts. As mentioned, the most suitable connection was version 3, in which we did not have to hold parts of the magnetic contacts in our hands; they were placed on a test device, and thus this eliminated the possible error rate of an incorrect reading of the detection distance. The implementation of the second phase took us almost three days with breaks, along with the fact that we only measured during the day. The given figure is diametrically different from the value of two and a half hours, which was how long the third phase lasted.

All phases followed each other and played an important role; if we did not identify in the first phase the most suitable way to perform the measurement, we would probably obtain skewed results and incorrectly estimate the value of the working distance needed for phase 3. Of course, it is possible to enter the working distance as we saw in the results in Table 2, the actual working distances are different than stated by the manufacturer. It is appropriate to consider whether in the case of setting the working distance to 25 mm in phase 3, we would obtain a significantly lower value of not activating the magnetic contact. This reasoning is appropriate, as the mentioned value is marginal according to the manufacturer.

In the third phase of testing, we completely automated the measurement of the reliability of magnetic contacts using a test device. To further define and understand the problem, we implemented 3 phases, as the results from the second and third phases are comparable. The difference occurs in the implementation of tests in terms of the magnetic field of the permanent magnet. In the case of manual tests, we moved the permanent magnet in a vertical position away from and to the tongue contact, while monitoring the value of the distance of its closing. In the second case of measurements, the movement of the permanent magnet was performed in the base at a horizontal level as it was mounted on a movable part that rotated about its axis. During all tests, the same conditions were maintained in terms of temperature and humidity in the room, and so the values of the magnetic field strength were the same. The difference between the measurements can be seen in several factors. In the case of manual measurement, we had to be maximally focused, and we needed to focus on the accuracy of the reading value; in the case of autonomous equipment, we eliminated these conditions, which reduced the measurement error as we assumed that within the working distance specified by the manufacturer, the magnetic contact should be able to work fully. From the point of view of the reliability of magnetic contacts, we can conclude that with the help of an automated test device, the value of the reliability of magnetic contacts reached 90% or 0.9 in the case of dimensionless expression [5,28,29].

The measurement of the detection characteristic, i.e., the closing and opening of the magnetic contacts, is an important and basic prerequisite for expressing the probability of the detector being overcome by the intruder or overcoming the secured zone. If the intruder wants to achieve the identification of the protected interest, it is necessary to cross all detection zones of the protected object. To express the overall probability of intruder detection, a cumulative probability is used that takes into account the number of zones in the building, the probability of correct detection by electrical security system components, the probability of system failure, the probability of correct information transmission, and the probability of human failure. The total value of the cumulative probability should be close to 1. Knowledge of the total value of the probability is also necessary due to the design of the system from an economic point of view [6,13,30,31].

The magnetic contacts, the measurement of which is devoted to the article, belong to the category of the probability of correct detection by the components of the electrical security system. We performed experimental measurements mainly due to a more accurate expression of the probability, as its decrease depends on several factors, and one of them is the reliability of the component. To subsequently express the total probability of the detection zone, the reliability of all components installed in the zone is calculated. Paradoxically, however, it can be stated that to express the total value of the probability of crossing the detection zone, it is sufficient to express the reliability of one component. In reality, however, the use of one component in a given zone is insufficient and their combination is recommended [7,20,32,33].

## 5. Conclusions

The article is devoted to a comprehensive, experimental testing of safety magnetic contacts from the point of view of the correct functionality and service life of a component intended for the mantle protection of objects. Magnetic contacts are one of the cheapest and most commonly used components of an electrical security system. The measurements are part of extensive scientific research activity to express the cumulative probability of detecting an intruder of a protected object. By their nature, they follow the reliability of passive infrared detectors, transmission systems, and other parts of the electrical security system.

We performed the experimental measurement in three phases. In the first we created three ways of recording the real working distance of magnetic contacts. In the second phase, we performed an extensive measurement for each magnetic contact using the most efficient method from the first phase. The final, third phase was focused on a large number of switching repetitions for one magnetic contact. As part of the results of the first phase, we found that the most effective way to record the working distance of magnetic contacts is a test circuit version 3, which consisted of a backup power supply, LED, resistor, and a structure made of nonconductive material. Using this method, we performed a total of 8000 repetitions in the second phase. With a higher number of repetitions, we were able to measure the values of the working distance almost identical to those stated by the manufacturer in the technical documentation, and therefore we can state that the magnetic contacts are reliable.

In the third phase, a test device was used which eliminated the error rate caused by the human factor. We could therefore clearly define the percentage of which the tested magnetic contacts are reliable because if the closing did not occur under the same conditions, it meant the failure of the magnetic contact. Such a failure means a malfunction of the security system, i.e., insufficient protection of the object, which may be endangered by the intruder. In addition to clearly defining the reliability of magnetic contacts, we improved the ability to obtain data by automating the testing process, as the testing itself does not require supervision and can be performed at any time and to any extent. Subsequently, we can better know the cumulative probability of intruder detection in a charming object as well as knowledge of the theoretical basis for simulation programs designed to find the most effective route of an intruder.

Experimental testing of magnetic contacts is complemented by long-term research into the reliability of components of the eclectic security system, and it is necessary to repeat it regularly. The disadvantage of measuring the reliability of these types of components is the time-consuming nature of the whole process and the possible error rate of the human factor when reading the value of closing or opening of the magnetic contact. However, these parameters can be largely eliminated utilizing a test device designed by us to test the lifetime of magnetic contacts. Although this device has several shortcomings, these can be eliminated, improved, and can become even more efficient in measuring the reliability of magnetic contacts in the future.

## Figures and Tables

**Table 1:** Results of measurements with a multimeter.

Type of Magnetic Contact	Datasheet Distance [mm]	Test Connection (Version 1)				Test Connection (Version 2)				Test Connection (Version 3)			
		Clamping Distance [mm]	Standard Deviation	Opening Distance [mm]	Standard Deviation	Clamping Distance [mm]	Standard Deviation	Opening Distance [mm]	Standard Deviation	Clamping Distance [mm]	Standard Deviation	Opening Distance [mm]	Standard Deviation
Bestkey BR1013	0–25	35	3.18	51	4.14	29.5	2.74	38.3	2.54	24	2.67	31	1.59
Bestkey BS2013	0–31	39	3.38	46	2.83	28	4.68	38	2.95	15.5	3.69	22.6	1.31
SUNWAVE SD 8561	12.7–25.4	24	4.28	46.5	4.05	28.5	4.81	38	4.48	28.1	1.67	36.1	2.53
USP 130SP	0–25	30.5	2.71	46.5	3.99	30	4.76	30	2.05	27.5	3.89	29	1.83

**Table 2:** Measured values at 18,000 repetitions.

Type of Magnetic Contact	Datasheet Distance (mm]	Clamping Distance (mm)	Standard Deviation	Opening Distance (mm)	Standard Deviation
Bestkey BR1013	0–25	23.5	1.99	25.5	2.01
Bestkey BS2013	0–31	27	1.72	29.5	1.86
SUNWAVE SD 8561	12.7–25.4	15.5	2.06	23.5	2.54
USP 130SP	0–25	22.5	1.47	25	1.69

**Table 3:** Measured values using an autonomous test device.

Type of Magnetic Contact	Datasheet Distance [mm]	Set Distance [mm]	Number of Measurements	Activation of Magnetic Contact	Nonactivation of Magnetic Contact
Bestkey BR1013	0–25	22.5	10,000	9001	999
Bestkey BS2013	0–31	28	10,000	9104	896
SUNWAVE SD 8561	12.7–25.4	17	10,000	8913	1087
USP 130SP	0–25	22.5	10,000	9031	969

## Data Availability

Data is contained within the article.
